# Social Determinants of Breast Cancer Screening among Married Women: A Cross-Sectional Study

**DOI:** 10.34172/jrhs.2020.02

**Published:** 2020-02-16

**Authors:** Atefeh Ghanbari, Pardis Rahmatpour, Narges Hosseini, Malahat Khalili

**Affiliations:** ^1^Social Determinants of Health Research Center, Guilan University of Medical Sciences, Rasht, Iran; ^2^PhD Candidate in Nursing, Iran University of Medical Sciences, Tehran, Iran; ^3^Department of Epidemiology and Biostatistics, School of Public Health, Kerman University of Medical Sciences, Kerman, Iran

**Keywords:** Breast cancer, Screening, Social determinants of health

## Abstract

**Background:** Regular cancer screening is the best way for early detection of breast cancer, but studies showed the low participation rates of screening in Iran. We aimed to determine breast cancer screening among married women and related factors in North of Iran.

**Study design:** A cross-sectional study.

**Methods:** This cross-sectional study was carried out from Jan to Mar 2017 among 1472 married women in an urban population in Rasht City, North of Iran. Data were collected using a questionnaire included socio-demographic information and breast cancer screening behaviors. Descriptive statistics, chi-square and logistic regression were used for data analyzing with SPSS.

**Results:** The mean age of women was 35.1 ±6.5 years. Majority of women never performed clinical breast examination (70.7%) and regular monthly breast self-examination (52.2%). Only women over 40 yr performed mammography. Mammography performance was associated with health insurance (OR=4.99; 95% CI: 1.10, 22.53) and family history (OR=1.60; 95% CI: 1.19, 2.19), clinical breast examination was associated with age of women (OR=2.87; 95% CI: 1.90, 4.32) and breast selfexamination was associated with age and occupation of women [OR=1.67; 95 % CI: 1.16, 2.39, OR=1.65; 95% CI: 1.19, 2.29) respectively].

**Conclusion:** The rate of breast cancer screening was low among married women. Therefore considering the structural and cultural barriers, effective health education is essential to reduce inequality and increase the efficiency of screening programs.

## Introduction

Breast cancer (BC) is the most common type of cancer among women^1^, Asia and Africa have a more rapidly rising incidence rate than North America and Europe^2^. Moreover, the incidence trend of BC is rising in Iranian women^3^. Breast cancer prevalence in women is 23.1 per 100,000 women in Iran^4^.

Health behaviors are a meaningful target for health promotion programs and policies^5^. In Iran, screening programs are recommended in order to BC cancer incidence and its burden^3^. Women in Iran are reluctant to screen for BC. Therefore, there is a great need to change this behavior in women^4^.

The early detection of BC improves survival in BC patients^6^. Some social determinants were associated with cancer screening behaviors. Education and health insurance coverage were reported as significant predictors of BC screening^7^.

Despite the emphasis on regular BCscreening, still results indicated little participation in such preventive behaviors^2,8,9^. Even, BSE as a simple and effective method to make women aware of BC and encourage them to be examined by physicians in early stages was not performed regularly and monthly among women^10^. There are several personal and socio-cultural barriers to perform BC screening among women including lack of knowledge, financial, cultural barriers, fear and previous negative experiences^11^. The social determinants of health (SDH) “are the economic and social conditions that shape the heath of individuals, communities and jurisdictions as a whole” and SDH reflect the quality of resources made available by society to its members^12^. They help to predict outcomes, identify vulnerabilities, and provide a starting point for addressing health as a social concept^13^.

In Iran, mammography for women 40 yr and older once every year, CBE for 20-40 yr old women every 3 years and yearly after olds and above age 40 yr by a health professional and BSE for 20 yr every month recommended by Iranian Ministry of Health and MedicalEdustion^14,15^.

Although the role of BC screening in Iran is considered significant, the screening procedures, including a physician's examination and mammograms, are not free of charge. Therefore, we aimed to determine BC screening among married women to investigate how social determinants of health influence women’s health choices and practices.

## Methods

This cross-sectional study was carried out from Jan to Mar 2017 among married women in an urban population in Rasht City, North of Iran.

### Study population

Participants were selected by cluster sampling based on 33 healthcare centers under the coverage of Guilan University of Medical Sciences, Northern Iran. Each healthcare center was considered as a cluster and random sampling of sub-clusters was conducted based on record numbers of households in healthcare centers’ files. In each cluster, proportional to population size, sampling was done.

The sample size was determined 1472 with 95% confidence interval with d= 0.02 and *P* =0.17. Overall, 1472 out of 1550 distributed questionnaires were completed and returned; the response rate was 94.9%. The questionnaire was filled out by trained female staff of healthcare centers during a standardized face-to-face interview. Overall, 1472 questionnaires were completed and included in final analysis. The inclusion criteria considered included being married, living in Rasht City, with no history of BC, physical and mental ability to participate in the study and able to communicate verbally during the interview.

### Instrument

A researcher-made questionnaire was used including three parts. The first part was about socio-demographic characteristics: age, marital status, and level of education, number of children, occupation, health insurance coverage and self-rated health status (“poor”-“moderate”-“good”- “very good”- “excellent”). The second part consisted of YES-NO question that “did you ever have BC screening such as breast self-examination (BSE) and clinical breast examination (CBE) and mammography “. In positive response, frequencies of performing all cancer-screening behaviors were recorded with four choices (every two years, 3-5 years, more than 5 years, I don’t remember). Finally, an open question was asked about the reasons why mammography was not performed by women over 40 yr of age.

The questionnaire was translated into Persian language. Following the translations conducted by an Iranian professor of English literature, a native bilingual English speaker translated it back into English. Content Validity Ratio (CVR) and Content Validity Index (CVI) of questionnaire was assessed by faculty members of community health nursing and midwifery (n=15). Mean scores of CVI and CVR were more than 0.80. Cronbach’s α coefficients were computed to evaluate reliability of questionnaire, which was 0.85.

### Statistical analysis

Data analysis was carried out using SPSS ver. 22 (Chicago, IL, USA). Descriptive statistics such as frequencies and percentage were used. Chi-square or Fischer's exact test statistical methods were used for analytical statistic. Logistic regression analyses using the enter method were used to assess the association of social determinants factors and BC screening behaviors. *P* -value less than 0.05 was considered as statistically significant.

### Ethical approval

Written informed consent was obtained from each subject following a detailed explanation of the objectives and protocol of the study, conducted in accordance with the ethical principles and approved by the Ethical Committee of Guilan University of Medical Sciences (Ethical code: 2930003514).

## Results

Mean participants were 35.1 ±6.5, (from 15 to 45 yr). The majority of women were housewives (86.7%) and were educated (93.4%), had no child or one child (56.9%) and had health insurance (91.1%). Over half of women (58%) rated their health status as “good”. Generally, majority of women never performed CBE (70.7%) and regular monthly BSE (52.2%). Among women over 40 yr, 32.6% performed mammography. Majority of women done these cancer screening behaviors, they had been screened for BC within the last 2 years (Mammography=64%, CBE= 70.5%).

Results from Chi-square test for association between social determinants factors and BC screening are shown in Table 1. Logistic regression analysis showed that among women over 40 yr, mammography performance was associated with health insurance coverage (OR= 4.99, 95% CI: 1.10, 22.53); CBE performance increased with age of women (OR= 2.87; 95% CI: 1.90, 4.32). Moreover,performing BSE increased with age and more frequently among employed women [OR=1.67; 95 % CI: 1.16, 2.39; OR=1.65; 95% CI: 1.19, 2.29, respectively] (Table 2).

**Table 1:** Chi-square test for associations between socio-demographic factors and breast cancer screening

**Variables**	**Breast self-examination,** **n=1472**			**Clinical breast examination,** **n=1472**			**Mammography screening,** **n=350**		
	**No** **n=769**	**Yes** **n=703**	***P*** **value**	**No** **n=1041**	**Yes** **n=431**	***P*** **value**	**No** **n=236**	**Yes** **n=114**	***P*** **value**
Age (yr)			0.001			0.0001			
<30	192	118		250	60		-	-	
30-35	223	210		315	118		-	-	
35-40	187	192		258	121		-	-	
≥40	167	183		218	132		236	114	
Number of children			0.012			0.071			0.271
≤1	461	377		608	230		60	23	
≥2	308	326		433	201		176	91	
Education level			0.201			0.072			0.251
Illiterate	54	43		67	30		33	14	
Schooling	509	450		698	261		165	75	
Academia	206	210		276	140		38	25	
Occupation			0.001			0.008			0.092
Housewife	692	584		918	358		208	93	
Employed	77	119		123	73		28	21	
Health insurance coverage			0.081			0.031			0.022
No	78	53		103	28		19	2	
Yes	691	650		1221	442		217	112	
Self- rated health status			0.483			0.214			0.483
Excellent	27	25		31	21		4	4	
Very good	87	102		128	61		18	11	
Good	452	402		614	240		133	57	
Moderate	186	159		248	97		74	36	
Family History			0.201			0.483			0.031
No	469	200		641	161		200	34	
Yes	300	503		400	270		36	80	

**Table 2:** Evaluation of Social determinants of breast cancer screening by logistic regression

**Variables**	**Mammography**		**Clinical breast examination**		**Breast self-examination**	
	**OR (CI 95%)**	***P*** **value**	**OR (CI 95%)**	***P*** **value**	**OR (CI 95%)**	***P*** **value**
Age (yr)						
<30	-		1.00		1.00	
30-35	-		1.59 (1.11, 2.28)	0.011	1.47 (1.08, 1.99)	0.011
35-40	-		2.07 (1.41, 3.04)	0.001	1.52 (1.09, 2.11)	0.011
≥40	-		2.87 (1.90, 4.32)	0.001	1.67 (1.16, 2.39)	0.005
Number of children						
≤1	1.00		1.00		1.00	
≥2	1.41 (0.80, 2.50)	0.221	0.95 (0.72, 1.24)	0.721	1.20 (0.94, 1.54)	0.131
Education level						
Illiterate	1.00		1.00		1.00	
Schooling	1.49 (0.69, 3.21)	0.301	1.05 (0.65, 1.71)	0.822	1.28 (0.82, 1.99)	0.271
Academia	1.73 (0.68, 4.40)	0.242	1.37 (0.81, 2.30)	0.232	1.29 (0.80, 2.09)	0.282
Occupation						
Housewife	1.00		1.00		1.00	
Employed	1.42 (0.70, 2.88)	0.332	1.19 (0.85, 1.68)	0.301	1.65 (1.19, 2.29)	0.003
Health insurance coverage						
No	1.00		1.00		1.00	
Yes	4.99 (1.10, 22.53)	0.031	1.36 (0.87, 2.12)	0.173	1.20 (0.83, 1.75)	0.322
Self- rated health status						
Poor	1.00		1.00		1.00	
Moderate	0.82 (0.12, 5.26)	0.832	1.28 (0.50, 3.26)	0.601	1.04 (0.42, 2.57)	0.923
Good	0.45 (0.10, 1.94)	0.281	0.85 (0.38, 1.91)	0.692	1.31 (0.60, 2.84)	0.492
Very good	0.35 (0.10, 1.23)	0.101	0.69 (0.32, 1.47)	0.341	1.00 (0.48, 2.08)	0.982
Excellent	0.46 (0.13, 1.60)	0.222	0.68 (0.31, 1.49)	0.341	0.98 (0.40, 2.07)	0.971
Family History						
No	1.00		1.00		1.00	
Yes	1.60 (1.19, 2.19)	0.022	1.26 (0.70, 2.10)	0.201	1.10 (0.91, 1.62)	0.323

The five reasons reported for not performing mammography by women over 40 yr were: “I worry about the mammography results” (48.7%), “I think that I have no problem” (30.1%), “mammography is an expensive method of screening” (13.1%), “doctors didn’t tell me about BC screening” (7.2%) and “mammography is painful” (0.8%).

## Discussion

Results of this study indicated the low participation rate in BC screening among married women living in Rasht City, northern Iran. This is consistent with prior studies in other countries; Turkish study on BC screening reported that only 10.6%, 25.0% and 10.2% of women had done mammography, CBE and BSE respectively^16^. Another study was conducted among Arab women in Qatar reported that 13.9%, 31.3% and 26.9% of women performed BSE monthly, CBE and mammogram respectively^17^. Previous studies in different provinces of Iran such as Kerman^8^, Ardabil^18^, Kurdistan^6^, Mazandaran^19^, Hamedan^20^ and Lorestan^21^ participation rate in BC screening program were low. Among them, the participation rate in BSE ranged from the lowest 10.2% in Mazandaran (only Babol city) to the highest 36.7% in Ardabil. The prevalence of CBE performance ranged from 6% in Lorestan to 20.7% in Mazandaran. Since Iranian women did not perform mammography regularly, so results of studies are not comparable.

Among women who referred to health‑care centers in Tehran City, only 6.7% had mammogram^2^. To increase the participation rate of BC screening, programs should be free, regardless of type of insurance and socio-economic conditions^22^. The low prevalence rates of mammography utilization may partially be explained by the high cost of this procedure. BSE is a simple, low-priced, secure, effective diagnostic procedure when compared with mammography and CBE and BSE should be taught to women; but this screening method is not an appropriate alternative to mammography and clinical examination.

In this study, mammography use was higher among women with health insurance and family history. Older women were more likely to have CBE than younger women were and performing BSE increased with age, family history and employment. The positive association between having health insurance and receiving mammogram suggests that insurance allows access to this procedure, due to its high cost. Similar to Farzaneh et al., study, health insurance and age were two important factors for BSE and mammography^18^. By increasing age of women^10,23^ and having health insurance^7,24^ the performance in BSE, CBE and the frequency of mammography had promoted. Increase in age was associated with 5% increase (per year increase in age) in mammography adherence for low-income and high-income women ^25^. Subjects with a positive family history of BC have a higher awareness level and knowledge of screening methods^10^. Socioeconomic injustice in BC screening by mammography decreases over time^26^.

In this study, reported perceived barriers to mammography included lack of awareness and fear of cost that were similar to concerns, which expressed by participants in other studies^11,27,28^.

Because of poor knowledge of BC screening, women need training programs to become sensitive^29^. It is important to raise awareness of BC and pay attention to its barriers to screening programs^28^. In order to make an informed decision, women need training programs ^30^. Diagnosis of women without screening, given the higher risk of developing the disease, is especially important^31^. These findings can be used by health policy makers to be used in financial optimization in high-cost consumers who sometimes show more complications^5^. Moreover, these findings can promote public health efforts to support health care system sustainability.

The generalizability of our study findings are limited to women of similar demographics and characteristics as those who agreed to participate. The main limitation of this study is that it is based on self-reported information. Another limitation is the lack of women's knowledge of screening methods. Further studies are needed to verify the validity of such self-reports against medical records. Strong aspects of our study were the large sample size, selection of sample size from all primary care centers of urban population in Rasht City.

## Conclusion

BC screening is an important cost-effective strategy, particularly in low- and middle-income countries. Women’s participation rate in BC screening is low. In addition, study findings indicated the important role of social determinants (such as health insurance and employment) in screening of breast cancer. Low knowledge is the main barrier for cancer screening, therefore, considering the structural, cultural barriers, effective community-based health education for reducing inequality in health care and increasing the efficiency of screening programs is essential. Providing efficient and timely screening services may increase the likelihood of receiving it.

## Acknowledgements

We would like to thank the social determinants of health research center affiliated Guilan University of Medical Sciences.

## Conflict of interest

The authors declare that there is no conflict of interests.

## Funding

In this research, we received financial support for data collection in community from social determinants of Health Research Center affiliated Guilan University of Medical Sciences.

## Highlights

- Regular cancer screening is the best way for early detection of breast cancer.
- In Iran, the women show reluctance to do breast cancer screening.
- Some of social determinants were associated with cancer screening behaviors.
- The rate of breast cancer screening was low among married Iranian women.
- Our findings shows the important role of social determinants (such as health insurance, employment and family history) in breast cancer screening.
